# Influence of Physical Activity on the Regulation of Disease of Elderly Persons with Metabolic Syndrome

**DOI:** 10.3390/ijerph18010275

**Published:** 2021-01-01

**Authors:** Lucija Stetic, Ivan Belcic, Goran Sporis, Leon Stetic, Nikola Starcevic

**Affiliations:** 1 Faculty of Kinesiology, University of Zagreb, 10000 Zagreb, Croatia; lucija.stetic@gmail.com (L.S.); goran.sporis@kif.hr (G.S.); nikola.starcevic@kif.hr (N.S.); 2 Sheffield Hallam University, Sheffield S1 1WB, UK; leonstetic@yahoo.com

**Keywords:** metabolic syndrome, hypertension, overweight, blood pressure reduction, insulin resistance, cardiovascular risk

## Abstract

Metabolic syndrome is a group of metabolic risk factors whose combination significantly contributes to the development of the risk of cardiovascular disease, diabetes, stroke, some cancers and is a clear indicator of morbidity rate. The aim of this study was to identify physical activity programs that can successfully influence the reduction of risk factors in metabolic syndrome of the elderly. Subjects were aged between 60 and 80 years, had three of five signs of metabolic syndrome, and were randomly divided into three groups of 20 subjects. The first group conducted a continuous cycling ergometer (55% VO_2_max), the second group a physical activity strength program and the third was a control group. Before and after the experimental treatment body composition, biochemical parameters, functional parameters, cardiovascular functions, metabolic and hematological system were determined. Significant differences between control and experimental groups were determined using MANOVA. The training effects of the experimental and control groups were determined using the ANOVA for repeated measurements with Bonfferoni correction. The results showed that a physical activity program of strength has a better effect on disease regulation in the elderly with metabolic syndrome than a moderate-intensity physical activity program which also has a significant change but in less variables.

## 1. Introduction

Chronic diseases are among the most common and costly health problems that can be prevented and controlled, and they represent the leading cause of death and disability in the world [1]. The most common chronic diseases can be treated preventively by preventing risk factors such as high blood pressure, high blood cholesterol, and being overweight or obese. In addition, unhealthy eating habits, physical (in)activity and psychosocial relationships should be addressed.

Metabolic syndrome, also called insulin resistance syndrome or syndrome X, is a group of characteristics that together increase the risk of developing type 2 diabetes and heart disease. These characteristics include obesity, high blood pressure, elevated blood sugar levels, and high triglycerides. Keeping body weight, sugar concentration in blood, cholesterol and triglyceride levels under control contributes to a longer life and reduces the risk of heart attack and stroke. It should be emphasized that the main cause of obesity is combined with extremely poor eating habits [2], the aging process, so the basic approach is weight reduction and increased physical activity. In people who belong to the group of obese people, metabolic syndrome occurs in 60% of cases compared to people who have a normal body weight. In population of normal body weight, metabolic syndrome has been reported in only 5% of cases [3]. Physical inactivity contributes to the development of obesity and modification of muscle sensitivity to insulin while aging causes a gradual loss of muscle mass and thus leads to an increase in subcutaneous adipose tissue especially in the abdomen, ultimately resulting in changes that easily increase insulin resistance [4]. In population with metabolic syndrome, a change in lifestyle is recommended as a start of treatment, in order to reduce the risk factors for metabolic syndrome. If metabolic abnormalities persist after these interventions, treatment should focus on the treatment of type 2 diabetes and cardiovascular risk factors [5]. Metabolic syndrome is thus most often caused by a poor lifestyle, a combination or individual causes that can be hereditary or acquired. Exposure to stress, irregular and improper diet, high percentage of carbohydrates in the diet (more than 60%), physical inactivity and lack of sleep are just some of the segments and lifestyles of modern mankind. It is believed that such a lifestyle contributes to the increasing incidence of metabolic syndrome in the developed world. More than 90% of people with diabetes suffer from type 2 diabetes which can cause serious complications, affecting the eyes, nervous system and kidneys.

NCEP definition 13 (National Institute of Health) Third Report of the National Cholesterol Education Program Expert Panel on Detection, Evaluation, and Treatment of High Blood Cholesterol in Adults (Adult Treatment Panel III) does not necessarily include insulin resistance or glucose intolerance, but includes at least three or more of the possible five criteria. Metabolic syndrome is a group of characteristics among which you do not have to have all the characteristics to have metabolic syndrome. However, a person with one characteristic probably has others as well. Most experts define metabolic syndrome as the presence of three or more characteristics:

- abdominal obesity: abdominal circumference in men >102 cm, in women >88 cm;
- serum triglyceride concentration >1.69 mmol/L;
- Serum HDL cholesterol <1.04 mmol/L in men, <1.29 mmol/L in women;
- high blood pressure ≥130/85 mmHg;
- serum glucose concentration “on an empty stomach” ≥6.1 mmol/L.

The first sign of metabolic syndrome is obesity. Characteristically, it is a central type of obesity where adipose tissue accumulates mainly around the abdomen and body mass index is higher than 25. Layers of adipose tissue on the abdomen act on increased secretion of fatty acids into the liver circulation and lead to an increase in blood fat concentration. People with type 2 diabetes, in combination with other metabolic syndrome factors, are at high risk for the possibility of developing coronary heart disease and the cardiovascular system in the future [6,7,8].

In the population with normal blood glucose tolerance, 10–15% of persons with metabolic syndrome were recorded, while in the population with impaired glucose intolerance 42–64% of persons with metabolic syndrome, and in the population of persons with type 2 diabetes as much as 78–84% persons with metabolic syndrome [9]. A sedentary lifestyle has a negative impact on a person’s health, so every two hours more spent watching television increases the risk of developing diabetes by 14% and increases obesity by 23% [10]. The effect of insulin on the cardiovascular system can be classified into three categories, whose work is disrupted under the influence of insulin resistance in metabolic syndrome [2]: Vasodilation of blood vessels, increased absorption of sodium in the kidneys and increased activity of the sympathetic system. Insulin loses its vasodilating effect on blood vessels and increases sodium absorption in the kidneys. As vasoconstriction of blood vessels occurs and volume increases, arterial hypertension occurs in metabolic syndrome [11,12]. The effect of insulin resistance on carbohydrate metabolism results in increased blood glucose concentration, and in physiological conditions insulin stimulates the entry of glucose into fat and muscle cells, and thus also affects glucose synthesis in the liver and kidneys. In people suffering from metabolic syndrome with the appearance of insulin resistance, these processes become weaker and weaker, so higher insulin concentrations are then required for the same effect [2]. In order for the pancreas to compensate for this condition, there must be an increase in insulin synthesis, so that disorders at the level of pancreatic cells will secrete less insulin and the result will be the development of diabetes and hyperglycemia [13,14]. Free fatty acids are released from the expanded mass of adipose tissue, and in the liver stimulate increased production of glucose, triglycerides, and the secretion of low-density lipoprotein. The higher prevalence of low-density lipoprotein LDL and the decrease in high-density lipoprotein HDL occur due to associated disorders of lipids, i.e., lipoproteins. In muscle, free fatty acids will induce a decrease in insulin sensitivity, thus inhibiting insulin-mediated glucose uptake, and this will result in increased accumulation of lipids in the form of triglycerides and decreased glycogen production. An increase in free fatty acids and circulating glucose will increase the secretion of insulin from the pancreas and thus lead to hyperinsulinemia. Hyperinsulinemia easily leads to increased sodium reabsorption and sympathetic nervous system activity, which contributes to increased blood pressure and increased levels of free fatty acid circulation [15]. Regular physical activity of moderate intensity results in a decrease in triglyceride concentration and an increase in HDL concentration [16]. Daily physical activity has a great influence on the concentration of LDL particles, it significantly affects the lipidogram. It also has a significant effect on increasing HDL levels, whose concentration increases mostly under the influence of moderate-intensity physical activity [16,17,18].

The goal of this study is to determine the impact and better efficacy of various moderate-intensity physical activity programs on disease regulation in the elderly with metabolic syndrome. It has been hypothesized that the experimental program of continuous physical activity of moderate intensity and the program of physical activity type strength have a positive effect on disease regulation in the elderly with metabolic syndrome, and that the physical activity strength program has a better effect on disease regulation in the elderly with metabolic syndrome than a program of continuous physical activity of moderate intensity.

## 2. Materials and Methods

### 2.1. Ethics Committee Approval

This study has been approved by the ethics committee of the Faculty of Kinesiology, University of Zagreb. Each subject consented to voluntarily participation, where a physician and cardiologist examination (progressive stress test) was conducted before the study, to determine if they could participate due to certain physical loads during experimental process of the study.

### 2.2. Subjects

The study was planned for 86 subjects but was conducted on a total sample of 60 subjects (equally 30 men and women), as 26 subjects didn’t provide permission to proceed with the experimental study after medical examination. Subjects were aged between 60 to 80 years and had three of five parameters of metabolic syndrome. All subjects were randomly divided into three groups osubjects with first experimental group (age 66.95 ± 5.98, height 159.28 ± 4.18 cm), second experimf 20 ental group (age 65.30 ± 5.36, height 161.29 ± 5.86 cm) and control group (age 66.45 ± 5.11, height 160.30 ± 5.40 cm). Criteria used to include and select respondents:

- persons of chronological age 60 to 80 years
- three out of five parameters of metabolic syndrome
- inactivity in any form of organized physical activity for at least 6 months before the start of experimental treatment
- inactivity other than physical activity programs (experimental groups I and II) in other programmed forms of physical activity.

Exclusion criteria from the study: persons with cardiovascular and respiratory diseases, persons in the phase of recovery from some form of acute or chronic diseases and persons in the process of rehabilitation from injuries.

### 2.3. Variable Sample

Anthropometric and morphological parameters

- Body height (BH)
- Body weight (BW)
- Body Mass Index (BMI)
- Total amount of water (TW)

Cardiovascular and respiratory functions

- Maximum oxygen uptake (VO2max)
- Systolic blood pressure (RRs)
- Diastolic blood pressure (RRd)

Metabolic/hematological system

- Total cholesterol (CH)
- HDL cholesterol (HDL)
- LDL cholesterol (LDL)
- Blood glucose concentration (GB)

### 2.4. Procedure

In the first part of the research, the morphological and anthropometric characteristics, metabolic and hematological system were measured. After the initial measurements, the research was continued by conducting a progressive load test. The test measures the effect of physical activity on heart function, which can assess whether there is coronary heart disease. The electrical activity of the heart is recorded on an electrocardiogram, blood pressure and respiration are measured, while the test is performed on a bicycle ergometer according to pre-established measurement protocols (standardized conditions), and the goal is to achieve a certain heart rate depending on the individual condition. The load on the bicycle ergometer is dosed in Watts and is also an ideal test for the elderly because there is less likelihood of injury, as the subject is in a sitting position. The test starts with an initial load of 50 W, and each minute the load increases by 20 W, with 90 pedal stroke revolutions per minute. If the subject feels chest pressure, difficulty moving or lack of oxygen during the test, the test should be stopped. The electrocardiogram is monitored at each time of testing, and is recorded at the beginning of testing, i.e., in the pre-exercise phase, at all stages of physical activity, and at the end. Each degree of load lasts one minute and based on the achieved load and other recorded factors, the test result can be normal or pathological, which requires further processing and examination by a Doctor of Medicine [19,20]. All subjects of the experimental groups wore heart rate monitors (Polar RS 400, Polar Electro Oy, Kempele, Finland) during the physical activity program to have constant heart rate control and to avoid health contraindications.

After initial measurements of morphological and anthropometric characteristics, the first experimental group was included in a 12-week training program in the form of a continuous cycling ergometer of moderate intensity (55% VO_2_max). For the first four weeks, subjects underwent physical activity lasting a total of 35 min, from 5 to 8 weeks physical activity lasted 40 min, and from 9 to 12 weeks physical activity lasted 50 min. Throughout all 12 weeks the physical activity on the bicycle ergometer was under a load of 55% VO_2_max.

Second experimental group was included in a strength physical activity program that also lasted 12 weeks. The program load was regulated by the number of repetitions depending on the period in which it is performed, so the subjects performed eight exercises which were performed from 1 to 4 weeks with 10 repetitions and a break of 1 min between exercises. From the 5th to the 8th week, the same exercises were performed for 12 repetitions with a break of 1.5 min between exercises, and from the 9th to the 12th week, the same exercises were performed for 15 repetitions with a break of 2 min between exercises. Exercises: squat without load, lying Superman, abs with bent legs, biceps curl with a stick (500 g), lying Superman (opposite arm/opposite leg), climbing to a height of 10 cm, lifting the contorted legs to the chest from lying down position, lifting on the toes.

The third was the control group which did not have organized physical activity, subjects only continued with their usual daily activities.

As all study participants were volunteers, they were allowed to withdraw from the experimental treatment at any time during the program, and before the start of the experimental program, they were shown in detail the benefits that this research brings to them.

### 2.5. Data Analysis

Data were processed by the statistical program Statistical Package for Social Sciences SPSS (v17.0, SPSS Inc., Chicago, IL, USA). For all variables in all measurements, the central and dispersive parameters were calculated: arithmetic mean (AM), standard deviation (SD), minimum (MIN), maximum (MAX) and range of results (RR). Significance of differences (*p* < 0.05) between control and experimental groups in the measured variables in the initial and final measurements will be determined by applying multivariate analysis of variance (MANOVA). The training effects of the experimental and control groups will be determined using a univariate analysis of variance (ANOVA) for repeated measurements with Bonfferoni correction.

## 3. Results

For the study purposes and before the start of the 12-week physical activity program, all subjects were divided into 3 groups and subjected to testing of certain parameters shown in Table 1, Table 2 and Table 3. As all subjects involved in the implementation of this study were classified suffering from metabolic syndrome, it is considered that their initial state will not differ statistically significantly from each other, as it was the case.

Table 4 and Table 5 show the differences in the initial and final measurements between the subjects in all three groups, into which the subjects were randomly assigned.

The only statistically significant difference (*p* = 0.00) was obtained in the variable maximum oxygen uptake (VO_2_max), between both experimental groups, and between the first experimental group and the control group (Table 4).

Statistically significant differences (*p* = 0.00) were obtained in the total amount of water (between the two experimental and between the second experimental and control group), maximum oxygen uptake (between all three groups). Also, differences were found in HDL cholesterol between the second experimental and control group (*p* = 0.00) and in blood glucose concentration (*p* = 0.00) between first experimental and control group (Table 4).

Body weight did not differ significantly between the tested groups, but differed within individual groups in final measurements, in the second experimental (*p* = 0.00) and control groups (*p* = 0.00). The first experimental group did not record significant changes. Body mass index was significantly different between the initial and final measurements in the second experimental group (*p* = 0.00) and the control group (*p* = 0.00), while in the first experimental group no significant difference was obtained, nor between the groups. A statistically significant difference was obtained in the total amount of water in the second experimental group (*p* = 0.01) and the control group (*p* = 0.00). Maximum oxygen uptake shows a significant difference in the final measurement between all three groups (*p* = 0.00) and within each group (*p* = 0.00). Systolic blood pressure showed a difference within all three individual groups of subjects, but no difference was found between the groups. In the first (*p* = 0.00) and second (*p* = 0.02) experimental groups, a difference in the initial systolic blood pressure measurement was obtained compared to the final measurement. A significant increase was also obtained in the control group. Diastolic blood pressure showed a significant difference only within the control group (*p* = 0.00), while no significant difference was found within the first and second experimental groups, nor between all three groups. Total blood cholesterol showed a statistically significant difference within all three individual groups of subjects (*p* = 0.00), but no difference was found between the groups. In the first and second experimental groups in the initial measurement, the total cholesterol was significantly reduced compared to the final measurement, while in the control group it was increased. HDL cholesterol (high-density lipoproteins in the blood) shows a significant difference (*p* = 0.00) within all three individual groups of subjects, but also a significant difference between the other experimental and control groups. LDL cholesterol (low-density lipoproteins in the blood) also showed a significant difference (*p* = 0.00) within all three individual groups of subjects, but not between groups. Blood glucose concentration shows a statistically significant difference within the first and second experimental groups, but also the difference between the first experimental and control groups. No difference was found within the control group. In the first and second experimental groups, a significant difference was obtained between the initial and final measurements (*p* = 0.00), while the same was not obtained in the control group.

## 4. Discussion

Lack of physical activity is one of the key reasons for the emergence of many diseases of the human body. Although in modern times it appears more and more in childhood, physical inactivity in the elderly results in the development of severe chronic diseases, and consequently death. A study by Bankoski et al. [21] showed that subjects without metabolic syndrome spend an average of 9.5 h (65% of the time) sitting, while subjects with metabolic syndrome spend a higher percentage of time sitting (67.3%). Also, individuals with metabolic syndrome had longer average sitting periods (17.7 vs. 16.7 min), and lower intensity during the sedentary period (14.8 vs. 15.8 average sums per minute), but also had smaller sitting breaks (82.3 vs. 86.7 min), which was adjusted for age and gender. According to the data of the authors Lovic et al. [22], subjects suffering from metabolic syndrome had a much higher percentage of recorded deaths of the cardiovascular type, while subjects without metabolic syndrome had a lower percentage, although the statistical difference was not significant, but therefore significantly higher hospitalizations and cardiac dysfunction factors in subjects suffering from metabolic syndrome. Physical activity is an extremely important factor in preserving the health and functional abilities of the elderly. In order for the elderly to be independent for as long as possible from the help of another person in carrying out daily life activities, it is necessary to maintain motor skills and especially muscle strength, flexibility and balance. One of the main elements for engaging in physical activity is motivation, as a limiting factor, which contributes to a lack of motivation and willingness to which allows an individual to regularly engage in a program of physical activity that will have a multiple impact on his physical and mental health. Most people cite lack of time and busyness engage in some form of physical activity. When a person reaches the moment when he decides to be actively involved in a physical activity, it usually happens once or twice a week, but in inappropriately increased intensity, which results in a large number of injuries, and thus cessation of activity [23]. Less than 20% of older males engage in some form of physical activity, while the female population is on average twice as likely not to engage in physical activity, especially when it comes to older women [24]. Overweight is more affected by reduced or no exercise than increased calorie intake, resulting today in more than 1.46 billion overweight adults [25]. It is very difficult to cause any form of change in the behavior of people, who for many years learn and use the principles of living that are harmful to their global health, and they cannot notice it until the moment when their health is threatened, and this is usually too late for changes. Habits needs to be changed in younger populations. Implementing a new healthy strategic plan and program at the global level is extremely difficult, if it is an elderly population, so favorable results of the World Health Organization strategy can be expected only when the younger population that has adopted some changes from an early age grows up [26].

All subjects who participated in this study belong to the group of overweight people, so statistically significant results (*p* < 0.05) were obtained in second experimental group that conducted programmed physical activity of strength type and under the influence of this activity subjects decreased body weight. The control group did not perform any form of physical activity and the subjects increased body weight in the final measurement. In the first experimental group, which performed programmed aerobic physical activity, no statistically significant difference in the body weight variable was found, meaning that strength program had a greater impact on weight loss. Similar results were obtained by a group of authors [27] and concluded that physical strength activity was associated with a decrease in visceral adipose tissue and an increase in muscle mass. Studies by several authors [28,29,30] have shown that physical activity has a large impact on weight loss, whether it is aerobic physical activity or strength based physical activity. In order to achieve a better result on weight reduction, it is necessary to combine different forms of physical activity that include aerobic and anaerobic forms, but also a reduction in caloric intake, and the result will be even more favorable. Because of genetic and metabolic differences between men and women, both in ability and in hormonal structure, the best recorded results of the effect of physical activity on weight reduction were observed in the overweight male population. [31]. Android or abdominal obesity is associated with the development of metabolic syndrome. People who are overweight are also more likely to develop several different cardiovascular diseases, so obesity is associated with an increased mortality rate, while visceral adipose tissue has an even stronger impact on its occurrence.

Overweight is considered as disorder of excessive caloric intake, i.e., excessive food intake, while researchers [32,33] suggest that the main reason for being overweight is insufficient energy consumption, i.e., insufficient physical activity. Programmed strength activities in second group resulted with significant effect on the subjects’ body mass index, which decreased, in contrast to aerobic programmed physical activity, which did not show significant differences, while the control group increased body mass index values. Both is logical due to physical inactivity in control group and with already mentioned results of body weight after treatment. Body mass index as one of the anthropometric measures and factors of metabolic syndrome, showing categorization of subject, is also closely related to other processes in the human body such as blood pressure. If there is an increase in body mass index, the prevalence increases hypertension. In men where the body mass index exceeds 30, the prevalence of hypertension increases to 24%, and in women where the body mass index exceeds 30, the prevalence of hypertension increases to 38% [34]. An increase in the mean value of the body mass index of 1.41 kg/m^2^ in men, and 1.31 kg/m^2^ in women will reduce life expectancy by one year [35]. In most western countries, there has been a large increase in the number of overweight people of 25–30%, i.e., people whose body mass index is more than 27 kg/m^2^, and it is believed that this number will grow more as no concrete measures have been taken, i.e., interventions to avoid the increasing prevalence of increasing the body mass index of the population. Increased body mass index highly affects the working capacity of the population and their general health. Such people fall into the category who will use the possibility of sick leave more intensively, burden the health system more, and thus will be less productive. The emergence of a modern lifestyle is one of the main factors in the prevalence of overweight people, whose activity is markedly reduced compared to the 1990s [32,36,37].

The value of the total amount of water shows whether a person is dehydrated, and the total amount of water in the body decreased in all three groups, which is not good, but only in the second experimental group and in the control group is significant. It follows that participants in this study (subjected to physical activity) were unlikely to increase fluid intake even though consumption was at a far higher level than it was usual for them. This is not the case for the control group where the decrease in total water amount can be attributed to an increase in body mass and body mass index. The total amount of water is the percentage of water in the body and speaks of proper and optimal fluid intake, so the normal total amount of water in the body varies in percentage from 45%–60%, and for men: 55%–65%. In people who regularly engage in physical activity, this percentage is approximately 5% higher than the stated ranges, precisely because these people contain a higher percentage of muscle mass and bones, and muscles contain a larger amount of water compared to adipose tissue [38].

A significant increase in maximal oxygen uptake was obtained in all groups, most notably in the group that performed the aerobic form of physical activity. Similar results were obtained by Stensvold et al. [39] when authors studied individuals with metabolic syndrome, and an increase in maximal oxygen uptake was recorded in the group performing aerobic physical activity and combined physical (strength and aerobic form) activity, while in other group of authors Thomas et al. [40] also obtained a statistically significant increase in maximal oxygen uptake during physical activity of walking/running in individuals with metabolic syndrome. Individuals with type 2 diabetes were subjected to a variety of physical activity programs, of which only a combined aerobic physical activity program recorded a significant increase in maximal oxygen uptake [30]. The strongest predictor of mortality in people with metabolic syndrome is low aerobic capacity, i.e., low maximum oxygen uptake. Activities lasting 30 min, carried out at 70% of maximum oxygen uptake, initially result in the consumption of glycogen from muscle about 50% energy, from blood glucose about 25% energy, and about 25% from body fat [41]. Maximum oxygen uptake is therefore the best indicator of cardiovascular readiness and aerobic endurance. Although overweight and aerobic capacity are two independent major predictors, i.e., factors for the development of fatal cardiovascular diseases, the association between aerobic capacity and mortality shows a stronger link, which suggests that development of aerobic capacity, is much more important than weight loss [42,43].

In obese people, hypertension occurs approximately six times more than in people with normal body weight, so there is correlation between an increase in body weight by 10 kg, which causes an increase in systolic pressure by 3 mmHg and diastolic pressure by 2.3 mmHg [34]. The first experimental group showed the highest impact on the reduction of systolic blood pressure. There was also a statistically significant decrease in the second experimental group, and an opposite increase in the control group (affected by the increase in body weight and body mass index). In a meta-analysis of 25 studies, a reduction in systolic blood pressure was found in 67% of subjects and diastolic blood pressure in 70% of subjects who performed regular physical activity, and the most pronounced effect of physical activity is observed in obese men [23]. In order to prevent the occurrence of metabolic syndrome, but also various other diseases, recommendation of Dikanovic [44] is 30 min of daily physical activity regularly every day, and this includes fast walking, running and cycling which should be combined with reduced caloric intake, i.e., diet which consists of various healthy foods rich in vitamins, minerals and fibers. The American Society for Sports Medicine [45] recommends 150 min of moderate physical activity per week to improve health in obese individuals. Arterial hypertension is the main independent factor of cardiovascular risk, and the risk of cardiovascular complications will depend on the severity of hypertension and its duration [23]. Physical activity reduces systolic blood pressure by an average of 6.9 mmHg in individuals with hypertension [46]. The aerobic form of physical activity has an effect on the reduction of systolic and diastolic pressure [47], and physical activity slows down the increase in arterial blood pressure in all age groups, especially groups that are in the aging process [48,49]. Aerobic form of physical activity as well as strength training influence lowering blood pressure in patients with diabetes [50]. The effect of different physical activity programs on diastolic blood pressure values was not recorded in this study. Only the statistical significance of the difference in the control group was obtained, where there was an increase in the value of diastolic blood pressure which can be attributed to inactivity. A large number of people suffering from hypertension and under the influence of certain therapies that cause a decrease in arterial blood pressure, after a certain period when they feel changes and stabilization of values, spontaneously discontinue the recommended therapy thinking that their arterial blood pressure values will remain at the same level. However, after a certain period, blood pressure values rise again and can cause various dangerous consequences for the organism if they are not addressed and controlled by specialists.

The reduction of total cholesterol and HDL cholesterol in the body is more influenced by programmed physical activity strength type than aerobic physical activity. The control group shows an increase in total, HDL and LDL cholesterol which is extremely bad for disease regulation and is a consequence of physical inactivity. The results of LDL cholesterol show that the aerobic form of physical activity has a more positive effect on the regulation of low-density lipoprotein than programmed physical activity of strength. The control group shows that inactivity has a significant negative effect on LDL cholesterol. An increase in blood cholesterol by 10% is manifested as an increase in the chances of developing coronary heart disease by as much as 20%, and this risk is further increased if a person is a smoker, has high arterial blood pressure, high blood glucose and low HDL cholesterol. The higher the values of these factors, the higher the risk of developing coronary heart disease [31]. HDL cholesterol has a protective role, and the higher it is, the less likely it is to develop cardiovascular disease.

The obtained results show that aerobic physical activity had a positive effect on the regulation of blood glucose concentration, as well as physical activity of strength, while in the control group no statistically significant difference were obtained. In subjects who were included in a two-week program of physical activity of an individual character and belonged to the group of insulin-independent character, there was an improvement in glucose regulation as well as progress in the variable of maximum oxygen uptake [51]. Dosed aerobic activity that is carried out systematically leads to an increase in the biological efficiency of insulin, where it has been reported that even after one workout there is an increase in sensitivity and the number of insulin receptors by 36% [31]. Insulin secretion in the body is controlled by blood glucose concentration, the effect of physical activity, and amino acids [51,52]. To reduce the incidence of type 2 diabetes, and to achieve better insulin sensitivity in diabetes, it is important to carry out physical activity that will have a beneficial effect on most factors that occur in metabolic syndrome. When a person with type 1 diabetes engages in regular physical activity, it is very likely that hypoglycemia will occur during the activity, especially in cases where the glycemic level was lower before the start of the activity. In physical activity before which glycemia indicated higher values, there will be an increase in glycemic values during physical activity [53].

Physical strength activity which has load aware that with in the aging process there are various changes in the human body, so or activity that has higher intensity than in physical endurance activity has shown a higher effect on muscle mass, where muscle contraction causes a positive effect on insulin, similar to achieved with glucose intake. Physical activity with resistance will have a beneficial effect on people suffering from metabolic syndrome, and on the regulation of their disease, since we are not only the amount of muscle mass but also metabolic functions.

Due to recruiting subjects as a convenience sample as all the subjects were from the same county and voluntarily accepted to participate in this research there is very little (almost unremarkable) possibility of impact on results and we can regard this as limitation of this study. But it is believed that subject sample of this study is a number wise enough and results have enough power to detect intervention effect in population of elderly people with metabolic syndrome. And this applies also to the strengths of the study which is believed are very good inclusion and exclusion criteria for subjects and also representation of metabolic syndrome parameters in the tested sample (Appendix A).

A regulated diet has a weaker effect on weight loss and maintaining the desired weight at the achieved level than is achieved by physical activity. Since a regulated or reduced diet affects the reduction of the metabolic part at rest, this is manifested in a reduction in the thermal effect of food. If we combine physical activity with reduced food intake, we will get an increase in the thermal effect of exercise, the metabolic part of resting energy use will increase, and after the activity (especially if it is high intensity) we will maintain lean body mass and increase energy consumption within 24 h. It is easier to reduce body weight and/or maintain it at the achieved level. After physical activity, there is an increase in the metabolic part of energy at rest and it remains elevated for the next three days, and it is an extremely important factor in weight loss [31]. Physical activity in people suffering from metabolic syndrome can be carried out under the supervision of a kinesiologist, if necessary, a Doctor of Medicine if it is a more severe case. The limiting factors for achieving faster and greater results is the disproportionate difference between body mass, joint structures, and the percentage of muscle mass, so a higher intensity of physical activity cannot be achieved. This causes a drop in motivation, but with professional help, all obstacles can be overcome and the necessary (desired) results can be achieved through hard work. People suffering from metabolic syndrome often think that due to caloric reduced food intake they will not have enough strength to meet the requirements of a physical activity program. It is also thought that genetic and metabolic factors limit progress in physical activity and weight loss, which greatly affects their final result. Therefore, professional help is needed to avoid this unsubstantiated opinion and adverse impact on the final result they strive for, which is the optimization of all relevant parameters that cause the metabolic syndrome.

## 5. Conclusions

The program of continuous physical activity of moderate intensity has a positive effect on the regulation of the disease in the elderly with metabolic syndrome. In particular, this refers to a significant improvement in the final measures compared to the initial one in maximum oxygen uptake, systolic blood pressure, total cholesterol, HDL cholesterol, LDL cholesterol, and glucose concentration.

The experimental strength program has a better effect than a continuous physical activity program on disease regulation in the elderly with metabolic syndrome. The results showed that a statistically significant difference was found between initial and final testing in body weight, body mass index, total amount of water, maximum oxygen uptake, systolic blood pressure, total cholesterol, HDL cholesterol, LDL cholesterol, and blood glucose concentration.

The results of the research advise people suffering from metabolic syndrome, as well as the elderly, to engage in any form of physical activity to increase their health status and to raise quality and duration of their life. This can be achieved with continuous physical activity program with moderate intensity and especially programmed strength training, which has a more effective impact on disease regulation and overall health. Inactivity in people with metabolic syndrome leads to poorer health and a higher degree of disease, or to a deterioration in the values that describe the status of people with metabolic syndrome. Physical inactivity endangers the already existing stage of the disease and can have significant contraindications to the organism, even with fatal consequences.

Due to voluntary application from all subjects for this study and also receiving good feedback from competent scientists in field of medicine and kinesiology, this study had a great and very important practical application for all the participants. Subjects have learned and practiced how to conduct physical activity from which they will only benefit and raise quality of their life to a better level, and consequently will prolong life expectancy itself.

## Figures and Tables

**Table 1:** Descriptive parameters of the 1st experimental group (initial and final).

	AM	MIN	MAX	RR	SD
AGE	66.95	60.00	77.00	17.00	5.98
BH	159.28	152.50	165.00	12.50	4.18
BW1	69.81	50.70	99.00	48.30	13.01
BW2	70.02	51.00	100.00	49.00	13.02
BMI1	26.92	21.10	36.80	15.70	4.35
BMI2	26.86	21.50	37.00	15.50	4.34
TW1	26.70	22.00	31.00	9.00	2.36
TW2	26.50	23.00	31.00	8.00	2.14
VO2max1	25.22	20.00	31.18	11.18	3.30
VO2max2	25.88	21.00	32.00	11.00	3.35
RRs1	139.23	107.00	178.00	71.00	21.28
RRs2	138.39	107.00	177.60	70.60	21.38
RRd1	86.73	67.00	119.00	52.00	13.30
RRd2	86.42	67.00	118.00	51.00	13.44
CH1	6.09	2.50	9.42	6.92	1.64
CH2	5.98	2.42	9.20	6.78	1.59
HDL1	1.31	1.20	1.43	0.23	0.06
HDL2	1.31	1.21	1.44	0.23	0.06
LDL1	3.42	3.21	3.62	0.41	0.11
LDL2	3.37	3.20	3.60	0.40	0.11
GB1	5.26	4.39	6.33	1.94	0.45
GB2	5.11	4.30	6.00	1.70	0.45

**Legend:** AM—Arithmetic mean, MIN—minimum value, MAX—maximum value, RR—range of results, SD—standard deviation, BH—body height, BW1—body weight 1. measurement, BW2—body weight 2. measurement, BMI1—body mass index 1. measurement, BMI2—body mass index 2. measurement, TW1—total amount of water 1. measurement, TW2—total amount of water 2. measurement, VO2max1—maximum oxygen uptake 1. measurement, VO2max2—maximum oxygen uptake 2. measurement, RRs1—systolic blood pressure 1. measurement, RRs2—systolic blood pressure 2. measurement, RRd1—diastolic blood pressure 1. measurement, RRd2—diastolic blood pressure 2. measurement, CH1—total cholesterol 1. measurement, CH2—total cholesterol 2. measurement, HDL1—high density lipoproteins 1. measurement, HDL2—high density lipoproteins 2. measurement, LDL1—low density lipoproteins 1st measurement, LDL2—low density lipoproteins 2. measurement, GB1—serum glucose concentration 1. measurement, GB2—blood glucose concentration 2. measurement.

**Table 2:** Descriptive parameters of the second experimental group (initial and final).

	AM	MIN	MAX	RR	SD
AGE	65.30	59.00	77.00	18.00	5.36
BH	161.29	153.50	175.00	21.50	5.86
BW1	69.90	57.00	90.00	33.00	8.95
BW2	68.96	55.87	89.00	33.13	8.96
BMI1	26.77	22.50	35.10	12.60	3.53
BMI2	26.48	22.00	34.89	12.89	3.53
TW1	26.35	20.00	30.00	10.00	2.28
TW2	25.55	19.00	29.00	10.00	2.24
VO2max1	22.55	17.00	27.00	10.00	2.74
VO2max2	22.85	17.45	27.00	9.55	2.72
RRs1	138.35	104.50	191.00	86.50	22.54
RRs2	137.69	103.00	189.00	86.00	22.38
RRd1	87.18	70.50	111.00	40.50	10.96
RRd2	85.90	68.00	109.00	41.00	11.43
CH1	5.64	3.99	7.11	3.12	1.02
CH2	5.53	4.00	7.00	3.00	1.02
HDL1	1.31	1.19	1.40	0.21	0.06
HDL2	1.33	1.23	1.43	0.20	0.06
LDL1	3.43	3.29	3.55	0.26	0.08
LDL2	3.41	3.28	3.52	0.24	0.07
GB1	5.64	4.67	6.61	1.94	0.63
GB2	5.49	4.58	6.48	1.90	0.59

**Legend**: Same as Table 1.

**Table 3:** Descriptive parameters of the control group (initial and final).

	AM	MIN	MAX	RR	SD
AGE	66.45	60.00	77.00	17.00	5.11
BH	160.30	151.00	172.50	21.50	5.40
BW1	65.98	54.00	94.50	40.50	11.10
BW2	67.05	55.00	95.00	40.00	10.97
BMI1	25.39	20.00	31.50	11.50	3.90
BMI2	25.89	21.00	31.70	10.70	3.76
TW1	25.25	19.00	29.00	10.00	2.15
TW2	23.75	19.00	27.00	8.00	2.05
VO2max1	21.30	17.00	26.00	9.00	2.54
VO2max2	20.25	17.00	25.00	8.00	2.43
RRs1	142.01	103.00	185.00	82.00	20.39
RRs2	143.17	105.00	185.90	80.90	20.21
RRd1	88.13	63.50	115.50	52.00	11.70
RRd2	88.44	64.00	115.89	51.89	11.58
CH1	5.84	3.64	8.26	4.62	1.36
CH2	5.94	3.79	8.50	4.71	1.36
HDL1	1.31	1.20	1.43	0.23	0.07
HDL2	1.30	1.19	1.42	0.23	0.07
LDL1	3.41	3.26	3.67	0.41	0.12
LDL2	3.42	3.25	3.68	0.43	0.12
GB1	5.66	4.70	7.61	2.91	0.84
GB2	5.72	4.80	7.80	3.00	0.86

**Legend**: Same as Table 1.

**Table 4:** Differences in all three groups in initial and final measurements.

	Initial Measurements		Final Measurements	
	f-Value	*p*-Value	f-Value	*p*-Value
AGE	0.47	0.62	0.47	0.62
BH	0.75	0.47	0.75	0.47
BWi	0.81	0.45	0.37	0.69
BMIi	0.92	0.40	0.32	0.73
TWi	2.23	0.12	8.50	0.00 ^b,c^
VO2maxi	9.67	0.00 ^a,b^	19.39	0.00 ^a,b,c^
RRsi	0.16	0.85	0.40	0.68
RRdi	0.07	0.93	0.24	0.79
CHi	0.53	0.59	0.69	0.50
HDLi	0.05	0.95	1.54	0.04 ^c^
LDLi	0.10	0.90	1.20	0.20
GBi	2.37	0.10	4.41	0.02 ^b^

^a^—differences between the two experimental groups. ^b^—differences between the 1st experimental and control group. ^c^—differences between the 2nd experimental and control group. **Legend**: BH—body height, BWi—initial body weight, BMIi—initial body mass index, TWi—initial total amount of water, VO2maxi—initial maximum oxygen uptake, RRsi—initial systolic blood pressure, RRdi—initial diastolic blood pressure, CHi—initial total cholesterol HDLi—initial high density lipoproteins, LDLi—initial low density lipoproteins, GBi—initial blood glucose concentration.

**Table 5:** Differences in initial and final measurements in both experimental and control group.

	*p*-Value (1 Group)	*p*-Value (2 Group)	*p*-Value (Control Group)
BW1	0.22	0.00	0.00
BW2			
BMI1	0.46	0.00	0.00
BMI2			
TW1	0.38	0.01	0.00
TW2			
VO2max1	0.00	0.00	0.00
VO2max2			
RRs1	0.00	0.02	0.00
RRs2			
RRd1	0.13	0.18	0.00
RRd2			
CH1	0.00	0.00	0.00
CH2			
HDL1	0.00	0.00	0.00
HDL2			
LDL1	0.00	0.00	0.00
LDL2			
GB1	0.00	0.00	0.27
GB2			

**Legend**: BW1—body weight 1. measurement, BW2—body weight 2. measurement, BMI1—body mass index 1. measurement, BMI2—body mass index 2. measurement, TW1—total amount of water 1. measurement, TW2—total amount of water 2. measurement, VO2max1—maximum oxygen uptake 1. measurement, VO2max2—maximum oxygen uptake 2. measurement, RRs1—systolic blood pressure 1. measurement, RRs2—systolic blood pressure 2. measurement, RRd1—diastolic blood pressure 1. measurement, RRd2—diastolic blood pressure 2. measurement, CH1—total cholesterol 1. measurement, CH2—total cholesterol 2. measurement, HDL1—high density lipoproteins 1. measurement, HDL2—high density lipoproteins 2. measurement, LDL1—low density lipoproteins 1. measurement, LDL2—low density lipoproteins 2. measurement, GB1—serum glucose concentration 1. measurement, GB2—blood glucose concentration 2. measurement.

## Data Availability

Data available on request due to privacy restrictions.

## Appendix A

**Table A1:** Representation of metabolic syndrome parameters in the tested sample.

#	AOC	STC	SHC	HYP	SGC	#	AOC	STC	SHC	HYP	SGC
1.	-	-	+	+	+	31.	+	+	+	+	+
2.	+	+	+	+	-	32.	+	+	+	+	-
3.	+	+	-	+	-	33.	+	+	-	-	+
4.	+	+	+	+	+	34.	-	+	+	+	+
5.	-	+	+	-	+	35.	+	+	+	+	+
6.	+	+	+	+	+	36.	-	+	-	+	+
7.	-	+	+	+	-	37.	+	+	+	-	-
8.	+	+	+	+	-	38.	+	+	+	-	-
9.	-	+	+	+	+	39.	+	+	-	+	+
10.	+	+	-	+	-	40.	+	+	+	+	+
11.	+	-	-	+	+	41.	+	+	-	-	+
12.	+	+	+	+	+	42.	-	+	-	+	+
13.	+	+	-	+	+	43.	-	+	+	+	-
14.	+	+	+	+	-	44.	+	+	+	+	+
15.	+	-	+	+	+	45.	+	-	+	+	+
16.	+	+	-	+	+	46.	+	+	+	+	+
17.	+	+	-	-	+	47.	+	-	+	+	+
18.	+	+	+	+	+	48.	+	+	+	+	+
19.	+	+	+	-	-	49.	+	-	+	+	-
20.	+	+	+	+	+	50.	+	+	-	+	-
21.	-	+	+	+	-	51.	+	+	+	+	+
22.	-	+	+	+	+	52.	+	+	-	-	+
23.	+	+	-	+	-	53.	-	-	+	+	+
24.	+	-	+	-	+	54.	+	+	+	+	-
25.	+	+	-	+	+	55.	+	-	-	+	+
26.	+	+	-	+	+	56.	+	-	-	+	+
27.	+	-	+	+	-	57.	+	-	+	+	-
28.	-	+	-	+	+	58.	+	+	+	+	-
29.	+	+	-	+	-	59.	+	+	-	+	+
30.	+	+	+	+	+	60.	+	+	+	-	-

**Legend**: AOC—Abdominal obesity measured as abdominal circumference in men > 102 cm, and women > 88 cm; STC—Serum triglyceride concentration > 1.69 mmol/L; SHC—Serum HDL cholesterol in men < 1.04 mmol/L, in women < 1.29 mmol/L; HYP—Hypertension ≥ 130/85; SGC—Serum glucose concentration “empty stomach” ≥ 6.1 mmol/L.
